# Feasibility of a Health Coach Intervention to Reduce Sitting Time and Improve Physical Functioning Among Breast Cancer Survivors: Pilot Intervention Study

**DOI:** 10.2196/49934

**Published:** 2023-12-19

**Authors:** Rowena M Tam, Rong W Zablocki, Chenyu Liu, Hari K Narayan, Loki Natarajan, Andrea Z LaCroix, Lindsay Dillon, Eleanna Sakoulas, Sheri J Hartman

**Affiliations:** 1 Herbert Wertheim School of Public Health and Human Longevity Science University of California San Diego La Jolla, CA United States; 2 Department of Pediatrics University of California San Diego La Jolla, CA United States; 3 UC San Diego Moores Cancer Center University of California San Diego La Jolla, CA United States

**Keywords:** physical function, sedentary behavior, quality of life, activPAL, health coaching, cancer survivors, physical functions, breast cancer, survivors, quality of life, sitting time, physical activity, walking, exercise, fatigue, sitting

## Abstract

**Background:**

Sedentary behavior among breast cancer survivors is associated with increased risk of poor physical function and worse quality of life. While moderate to vigorous physical activity can improve outcomes for cancer survivors, many are unable to engage in that intensity of physical activity. Decreasing sitting time may be a more feasible behavioral target to potentially mitigate the impact of cancer and its treatments.

**Objective:**

The purpose of this study was to investigate the feasibility and preliminary impact of an intervention to reduce sitting time on changes to physical function and quality of life in breast cancer survivors, from baseline to a 3-month follow-up.

**Methods:**

Female breast cancer survivors with self-reported difficulties with physical function received one-on-one, in-person personalized health coaching sessions aimed at reducing sitting time. At baseline and follow-up, participants wore the activPAL (thigh-worn accelerometer; PAL Technologies) for 3 months and completed physical function tests (4-Meter Walk Test, Timed Up and Go, and 30-Second Chair Stand) and Patient-Reported Outcomes Measurement Information System (PROMIS) self-reported outcomes. Changes in physical function and sedentary behavior outcomes were assessed by linear mixed models.

**Results:**

On average, participants (n=20) were aged 64.5 (SD 9.4) years; had a BMI of 30.4 (SD 4.5) kg/m^2^; and identified as Black or African American (n=3, 15%), Hispanic or Latina (n=4, 20%), and non-Hispanic White (n=14, 55%). Average time since diagnosis was 5.8 (SD 2.2) years with participants receiving chemotherapy (n=8, 40%), radiotherapy (n=18, 90%), or endocrine therapy (n=17, 85%). The intervention led to significant reductions in sitting time: activPAL average daily sitting time decreased from 645.7 (SD 72.4) to 532.7 (SD 142.1; β=–112.9; *P*=.001) minutes and average daily long sitting bouts (bout length ≥20 min) decreased from 468.3 (SD 94.9) to 366.9 (SD 150.4; β=–101.4; *P*=.002) minutes. All physical function tests had significant improvements: on average, 4-Meter Walk Test performance decreased from 4.23 (SD 0.95) to 3.61 (SD 2.53; β=–.63; *P*=.002) seconds, Timed Up and Go performance decreased from 10.30 (SD 3.32) to 8.84 (SD 1.58; β=–1.46; *P*=.003) seconds, and 30-Second Chair Stand performance increased from 9.75 (SD 2.81) to 13.20 completions (SD 2.53; β=3.45; *P*<.001). PROMIS self-reported physical function score improved from 44.59 (SD 4.40) to 47.12 (SD 5.68; β=2.53; *P*=.05) and average fatigue decreased from 52.51 (SD 10.38) to 47.73 (SD 8.43; β=–4.78; *P*=.02).

**Conclusions:**

This 3-month pilot study suggests that decreasing time spent sitting may be helpful for breast cancer survivors experiencing difficulties with physical function and fatigue. Reducing sitting time is a novel and potentially more feasible approach to improving health and quality of life in cancer survivors.

## Introduction

### Background

As there are over 4 million female breast cancer survivors in the United States, with numbers increasing yearly [1], finding strategies to improve physical function and overall survivorship quality of life is a paramount public health issue. Behavioral interventions to improve breast cancer survivors’ physical function and quality of life have typically focused on physical activity and increasing minutes of moderate to vigorous physical activity (MVPA) [2]. Despite the effectiveness of increasing physical activity to improve physical function and quality of life [3,4], not all survivors are able to make these behavioral changes. In particular, some breast cancer survivors have poor physical function that would make achieving the recommended level of MVPA [5] an unrealistic and potentially unsafe goal. Focusing on decreasing sedentary behaviors, such as prolonged sitting time, may be a more appropriate and attainable behavioral target for breast cancer survivors with worse physical health.

Sedentary behavior among breast cancer survivors is associated with increased risk of cancer recurrence, lower quality of life, and premature mortality [6-8]. Sedentary behavior is any waking behavior done in a sitting, reclining, or supine position and characterized as an energy expenditure ≤1.5 metabolic equivalents [9]. Cancer survivors spend over 9 hours a day being sedentary and are more sedentary than individuals without a cancer history [10-12]. Among breast cancer survivors, long sitting bouts (≥20 min in duration) are associated with worse physical function [1,13,14] and lower quality of life [15].

Sedentary behaviors such as sitting and reclining result in decreased muscle activation and are associated with sarcopenia and subsequent physical and functional decline [9,11,16]. Decreasing sitting time has been shown to be effective in increasing postural muscle activation with improved physical and mental health benefits [17-19]. However, there is limited research on decreasing sitting time in breast cancer survivors; most studies on sedentary behaviors have used combined sedentary and physical activity interventions [20,21]. Furthermore, it is unknown what impact reducing sitting time has on physical function and quality of life of cancer survivors [22-26]. Given the growing number of breast cancer survivors with physical function limitations affecting their quality of life, there is a pressing need to develop effective and feasible sedentary behavior interventions. Therefore, we designed Rise, a 3-month, theory-based intervention aimed to reduce sitting time and improve physical function and quality of life among female breast cancer survivors with physical function limitations.

### Objectives

The primary aim of this pilot study was to determine the feasibility of enrolling and retaining breast cancer survivors who reported some physical function limitations into a 3-month intervention to reduce sitting time. The secondary aim was to investigate if the intervention could reduce objectively measured sitting time via activPAL (a thigh-worn accelerometer; PAL Technologies). The tertiary aim was to examine the preliminary impacts of the intervention on objectively and self-reported physical function and multiple aspects of quality of life from baseline to 3 months. We also solicited qualitative participant feedback on the Rise intervention and suggestions for future improvements.

## Methods

### Participants and Design

Participants were recruited between February and May 2022, from individuals who agreed to be contacted for future research studies and from those who were not eligible for an ongoing physical activity intervention trial [27]. Trained recruiters described this study’s activities and confirmed eligibility over the phone before potential participants were scheduled for their first in-person study visit. The target enrollment (n=20) was based on available funding. All participants provided written informed consent. Participants were then enrolled in a 1-arm feasibility trial of a 3-month intervention to reduce sitting time. The trial was registered with ClinicalTrials.gov (NCT05260723). Data were collected from February through August 2022 in San Diego, California, United States.

### Eligibility

Eligible women (1) were breast cancer survivors diagnosed at stages 1-4, (2) received chemotherapy, radiation, immunotherapy, or endocrine therapy as part of their breast cancer treatments, (3) were at least 1 year after active treatment (eg, chemotherapy), (4) were sedentary (defined as 7 h or more of sitting time per d on at least 4 d as measured by the activPAL), and (5) had a T-score of less than 50 on the Patient-Reported Outcomes Measurement Information System (PROMIS) physical function measure. Exclusion criteria were (1) medical condition that interferes with ability to safely stand or stay balanced, (2) other cancer diagnosis that occurred after their breast cancer diagnosis, (3) stage 4 breast cancer with brain metastases or less than 12 months life expectancy, and (4) unable to commit to a 3-month study.

### Ethical Considerations

The University of California, San Diego institutional review board approved all study procedures (IRB # 171548). Informed consent and the ability of participants to opt out were provided to all participants. All data were stored on a secure HIPAA (Health Insurance Portability and Accountability Act)–compliant database, REDCap (Research Electronic Data Capture; Vanderbilt University), at the University of California, San Diego [28]. Participants received a US $25 gift card for completing baseline measures and a US $50 gift card for the 3-month final assessment.

### Measurement Procedures

Interested and eligible women were scheduled and consented. At the in-person baseline visit, height and weight were taken and three physical function tests were completed: (1) 4-Meter Walk Test, (2) Timed Up and Go (TUG), and (3) 30-Second Chair Stand. At the end of the baseline visit, participants were given a thigh-worn accelerometer (activPAL) to measure sedentary behaviors and a hip-worn accelerometer (ActiGraph GT3X+; ActiGraph LCC) to measure physical activity. Participants were asked to wear both devices for 24 hours continuously for 7 days and to bring the devices to the second visit. Between the baseline and second visits, participants also completed web-based surveys, including self-reported measures of quality of life. At the second visit, data from the activPAL were screened for sitting time eligibility, which was a minimum of 4 days of wear with greater than 7 h/d of total sitting time on >50% (n=4) of days worn. Participants who met all the eligibility criteria were then started in the Rise intervention. All baseline measures were repeated at the 3-month final assessment.

### Intervention

The Rise intervention consisted of 7 individual, personalized health coaching sessions over the course of 3 months. The intervention was delivered by 2 health coaches trained in motivational interviewing. The 5 in-person sessions were 60 minutes each (weeks 1, 2, 3, 4, and 8) and conducted at University of California, San Diego Moores Cancer Center in La Jolla, California, while the two 30-minute sessions (weeks 6 and 11) were conducted remotely via telephone or Zoom (Zoom Video Communications) per the participant’s preference. Intervention topics were modeled from a sitting less intervention aimed to reduce sitting time in postmenopausal women [29] and adapted for breast cancer survivors. Adaptations included modifying the educational materials and health coach sessions to reflect how the goal of decreasing sitting time may improve health-outcomes and minimizes the risk of cancer recurrence [20].

**Figure 1:**
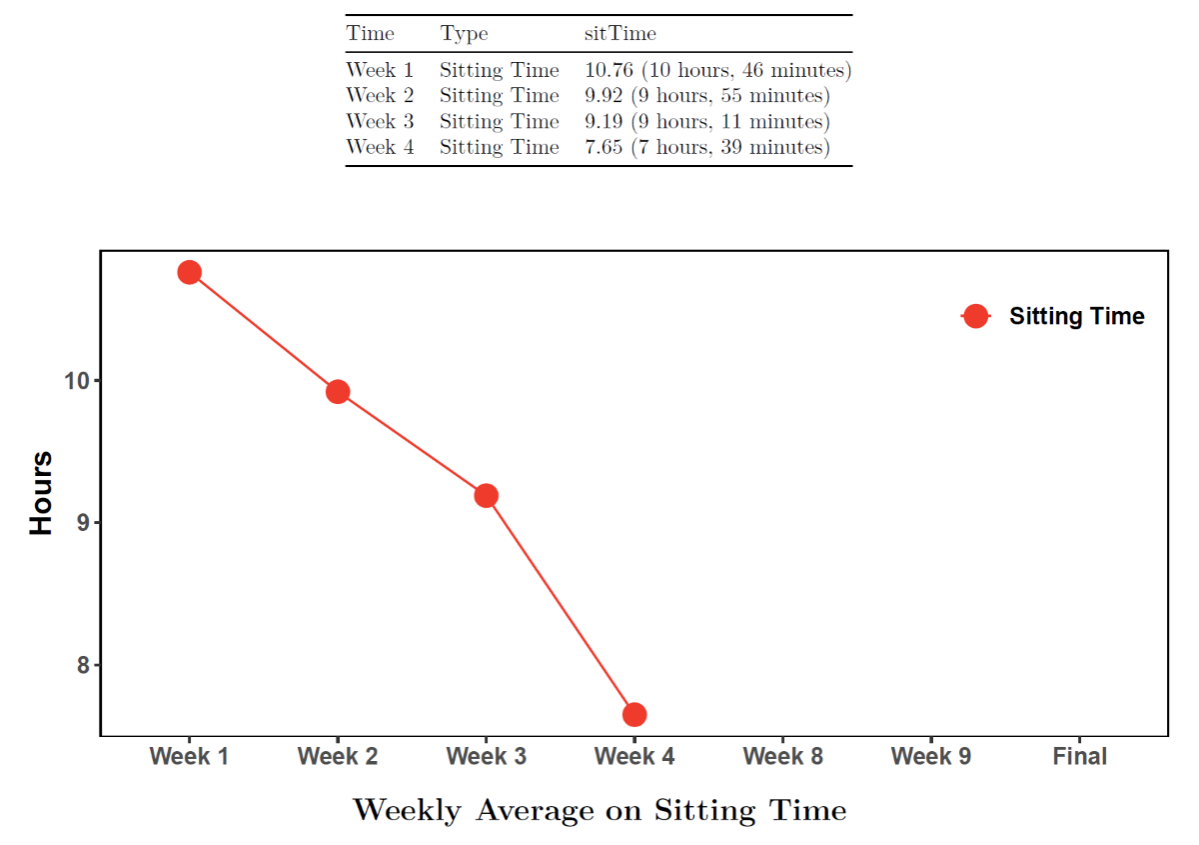
Sample feedback graph from a participant’s activPAL data of their average weekly change in sitting time.

**Figure 2:**
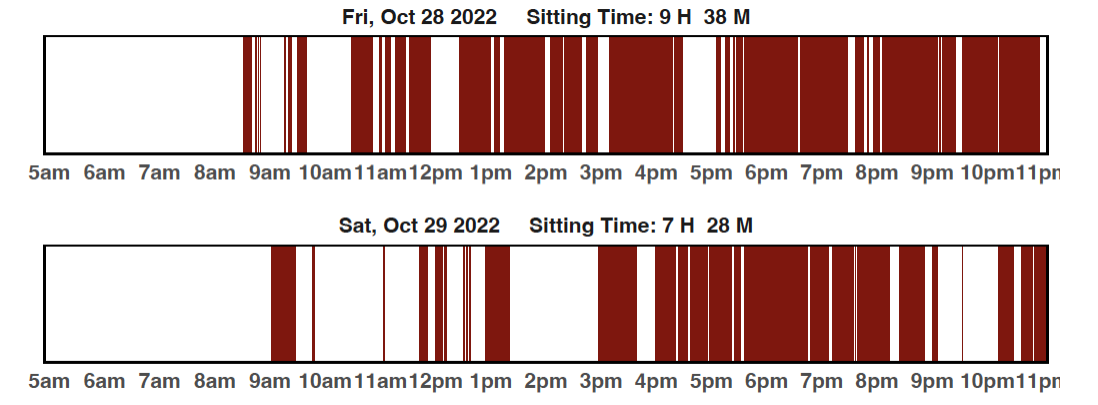
Sample feedback graphs from a participant’s day-level activPAL data. Red indicated sitting occurred and white bars indicated upright positions (eg, standing and walking).

The sitting less intervention components are based on habit formation [30-33] and the social cognitive theory [34], which can be mapped to behavioral strategies found to be important by Michie et al [35]. Sitting is a highly automatic behavior and breaking it up requires conscious recognition to promote the formation of different habits [36]. Unlike traditional physical activity interventions, where participants may be able to plan a walk into their day and track physical activity at the daily level with a pedometer, reducing and interrupting prolonged sitting requires more intense self-monitoring and specific goal-oriented feedback [34]. Particularly important are prompts and environmental cues to continually help participants become more conscious of sitting behaviors [37]. As part of the Rise intervention, participants were asked to wear the activPAL on their thigh continuously for weeks 1-4 and again during week 7 to receive feedback on their sitting time (Figures 1 and 2) to promote self-monitoring to support habit formation. Figure 1 is an example feedback report provided to participants. It shows the average sitting time each week they wore the activPAL so that participants can see how their sitting time changes across the intervention. Participants were encouraged to gradually reduce daily sitting time to achieve a 120-minute reduction in sitting time per day from their baseline. Figure 2 is an example feedback report provided to each participant that shows their day-level activPAL data. The red bars indicated their sitting time and white bars indicate when they were in an upright position (eg, standing and walking). Waking and sleep time were adjusted for and displayed as complete white sections to the left of the first red bar. Using both activPAL graphs, the health coach supported participants to set goals with a specific action plan for the upcoming weeks. To further support behavior change, a variety of prompts and environmental cues were provided, including a standing desk or table, timer, cue cards, and a wrist-worn device (ie, Lintelek watch) to prompt breaks from sitting.

### Measures

#### Feasibility

Feasibility was measured via the recruitment, adherence, and retention outcomes. Adherence and retention rates were measured as the percentage of participants that (1) completed all 7 health coach sessions and (2) completed the 3-month final assessment.

#### Objective Measure of Sedentary and Physical Activity Behaviors

The activPAL, a triaxial thigh-worn accelerometer, was used to objectively measure sedentary behaviors and stepping pattern at baseline and 3-month assessments. Event files from the activPAL were extracted via the CREA classification algorithm (version 8; PALanalysis), which was set to require ≥4 second for a new posture to be registered and generated sleeping time for removal from analysis. Minutes spent in various sedentary behaviors (ie, sitting, standing, sit to stand transitions, and stepping time) were derived from continuously recorded data [38,39]. The activPAL has been validated with good reliability and validity [40-42] for measuring sedentary behavior and stepping pattern in community-dwelling older adults [43].

The ActiGraph GT3X+, a triaxial hip-worn research grade accelerometer, was used to objectively measure MVPA minutes. Sufficient ActiGraph wear time was classified as at least 5 days with 600 minutes (10 h/d) or 3000 minutes (50 h) across 4 days. Wear time validation was analyzed via Choi et al [44] 2011 guidelines and processed with ActiLife software (ActiGraph LCC). ActiGraph data were processed with low frequency extension and aggregated to 60-second epochs via established Freedson et al [45] MVPA cutoff points defined as 1952 or more counts per minute (3.00-7.00 metabolic equivalents). ActiGraph has been validated [46] with good reliability [47] for measuring MVPA in adults under free-living conditions.

#### Physical Function Outcomes

Objective physical function outcomes were measured with the 4-Meter Walk Test, TUG, and 30-Second Chair Stand. The 4-Meter Walk Test consisted of measuring their normal walking pace for 4 meters, with the time recorded from when they began walking to when the first foot crossed the 4-meter line. Participants performed the test twice and the faster time was used. This measure of gait speed has excellent interrater, intrarater, and test-retest reliability and convergent validity among community-dwelling older adults [48]. The TUG measured the amount of time it took to get up from a chair, walk 3 m down a path, turn 180 degrees around a cone, walk back, and sit down. Participants performed the test twice and the faster time was used. It has established validity and test-retest reliability in older cancer survivors [49]. The 30-Second Chair Stand measured how many full sit-to-stand repetitions the participant completed in 30 seconds. Participants performed the test once. It has excellent interrater and test-retest reliability and criterion validity in community-dwelling older adults [50]. Self-reported physical function was measured using the PROMIS Physical Function scale. This measure uses computer adaptive testing, which was developed to measure a full range of functions, minimizing ceiling and floor effects [51].

#### Quality of Life Outcomes

Depression, anxiety, fatigue, sleep, and pain were assessed through the PROMIS cancer scales for depression, anxiety, fatigue, sleep, and pain interference that were developed for cancer survivors and are administered using computer adaptive testing [52,53]. These measures have been shown to be responsive to intervention and prospective studies in cancer survivors [54,55].

#### Intervention Feedback

The acceptability of the intervention was assessed via web-based satisfaction surveys regarding various components of the Rise intervention, barriers and facilitators outside of the program contributing to reducing sitting time, and satisfaction with the intervention tools. Satisfaction with the intervention tools was rated on a Likert-type response covering topics about how helpful the features from the intervention were on a 5-point scale ranging from 1 (not at all helpful) to 5 (extremely helpful) and how much they liked the features ranging from 1 (disliked a lot) to 5 (liked a lot). Barriers and facilitators contributing to reducing sitting time and various components of Rise were gathered via both closed and open-ended written questions to assess parts of the program that participants felt were the most and least helpful in reducing their sitting time. Open-ended written questions also asked about ways to improve the intervention and to better address the needs of cancer survivors.

### Statistical Analysis

Descriptive statistics were used to describe participant demographics and breast cancer characteristics. Except where stated otherwise, continuous variables were presented in mean (SD), categorical variables were presented as number (n) and percent (%), and the statistical type I error (α-level) was set at .05. Feasibility was calculated as the percentage of participants that (1) completed all 7n health coach sessions and (2) completed the 3-month final assessment compared to the baseline enrollment (n=20). Linear mixed models (LMM) with participant-level random intercept were fitted by repeated measures of outcome and fixed effects of the visit. LMM analyses were performed to investigate the intervention effect on physical function, PROMIS, sedentary behavior, and physical activity measures from baseline to the 3-month assessment. The coefficient (β) is an estimation of intervention effect from the baseline to the 3-month visit. All analyses were performed in R statistical programming (R Foundation for Statistical Computing) [56] language and LMM was implemented in R package *nlme* [57,58].

## Results

### Participant Characteristics

The participants’ average age was 64.5 (SD 9.4; range 51-78.3) years, and their BMI averaged 30.4 (SD 4.5; range 22.4-38.0) kg/m^2^. In total, 15% (n=3) of the participants identified as Black, 20% (n=4) as Hispanic or Latina, and 55% (n=14) as non-Hispanic White, with 60% (n=12) having a college degree or higher. On average, time since diagnosis was 5.8 (SD 2.2) years with 40% (n=8) treated with chemotherapy, 90% (n=18) having received radiation, and 85% (n=17) were prescribed endocrine hormone therapy (see Table 1 for complete descriptive statistics).

**Table 1:** Participant demographics (n=20).

Characteristics		Value
Age (y), mean (SD)		64.5 (9.4)
BMI (kg/m^2^), mean (SD)		30.4 (4.5)
**Education, n (%)**		
	Some college or less	8 (40)
	College graduate	5 (25)
	Graduate degree	7 (35)
**Marital status, n (%)**		
	Divorced or separated or widowed	10 (50)
	Living with partner	8 (40)
	Never married	2 (10)
**Ethnicity, n (%)**		
	Hispanic or Latina	4 (20)
	Non-Hispanic or Latina	16 (80)
**Race, n (%)**		
	Black	3 (15)
	White	14 (55)
	More than 1 race	2 (10)
	Other	1 (20)
**Cancer stage, n (%)**		
	Stage 1	12 (50)
	Stage 2	6 (30)
	Stage 3	1 (5)
	Stage 4	1 (5)
**Hormone therapy, n (%)**		
	Currently taking	8 (40)
	Previously took	9 (45)
	Not prescribed	3 (15)
**Surgery type, n (%)**		
	Lumpectomy	15 (75)
	Mastectomy	5 (25)
Time since diagnosis (y), mean (SD)		5.8 (2.2)
Received chemotherapy, n (%)		8 (40)
Received radiation, n (%)		18 (90)

### Enrollment and Feasibility

**Figure 3:**
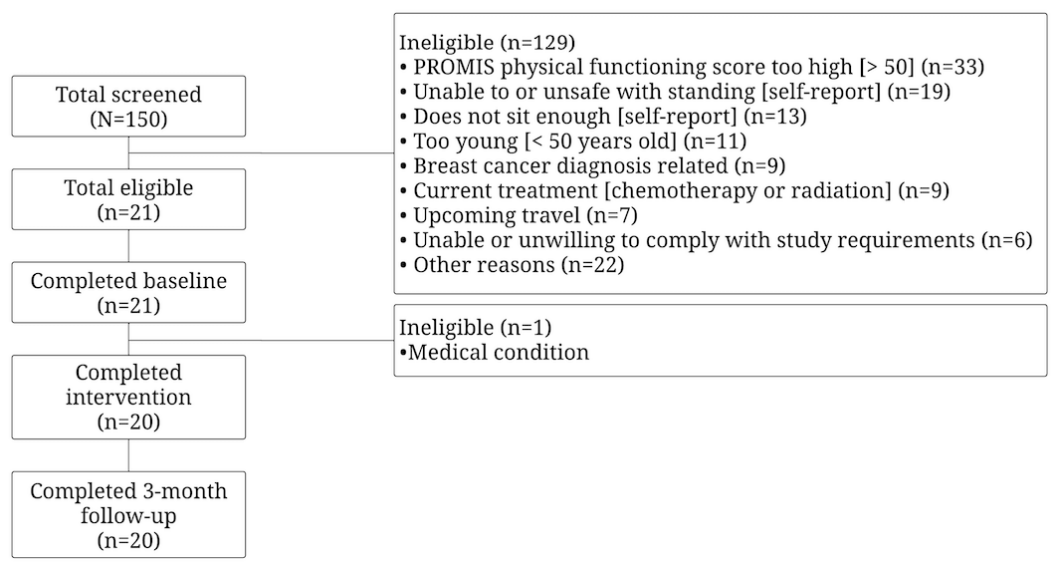
CONSORT (Consolidated Standards of Reporting Trials) diagram. PROMIS: Patient-Reported Outcomes Measurement Information System.

Participants were predominantly recruited via previous research study lists and telephone-screened to determine eligibility. The CONSORT (Consolidated Standards of Reporting Trials) diagram (Figure 3) showed that out of 150 women who were screened for eligibility, 21 were eligible and enrolled into this study. The most common ineligibility reasons included PROMIS physical functioning score being too high (>50; n=33), being unable to or unsafe when standing (n=19), and self-reported not enough time spent sitting (n=13). At the baseline visit, 21 women were deemed eligible. However, an a priori decision was made to exclude 1 participant from analyses due to a heart attack that occurred 2 weeks into this study. The final data set for all analyses includes 20 participants. Adherence to this study was high; all 20 participants completed all 7 intervention sessions and the 3-month final assessment resulting in a 100% (n=20) retention rate.

### Changes in Sedentary Behaviors and Physical Activity

**Figure 4:**
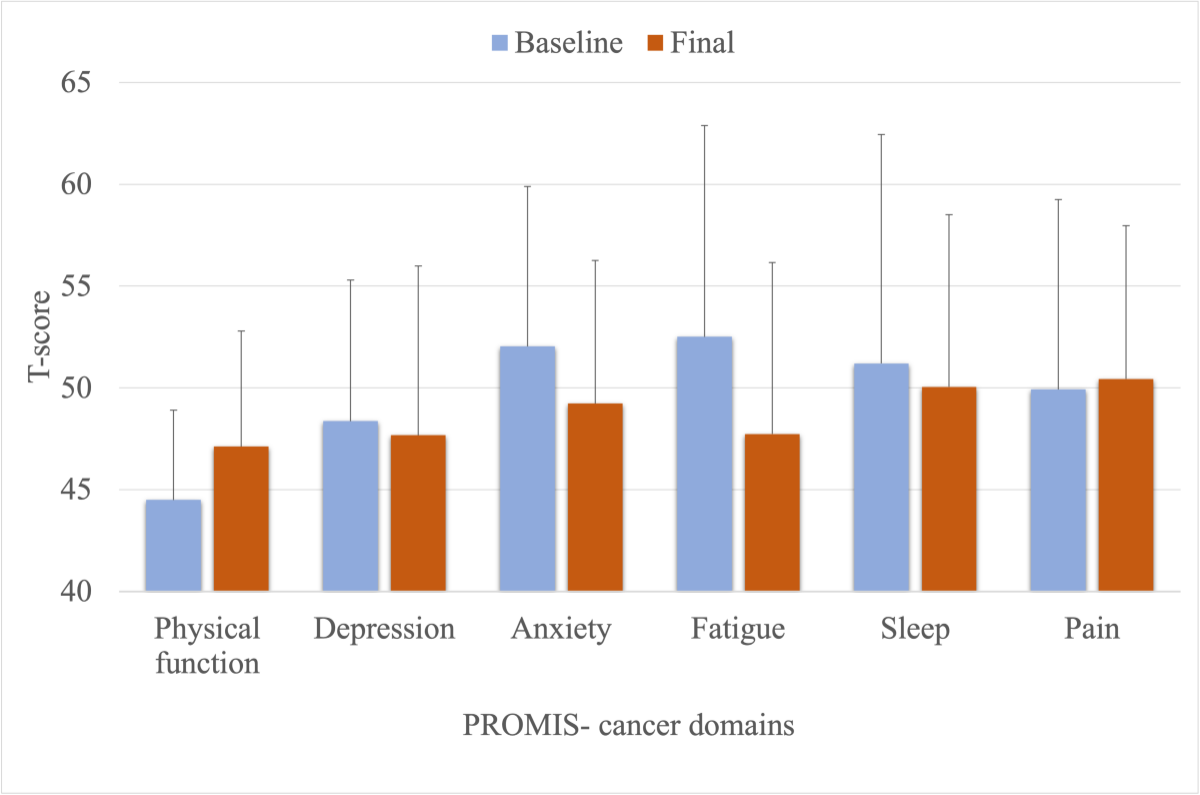
Quality of life outcomes (mean and SD were presented by visit). PROMIS: Patient-Reported Outcomes Measurement Information System.

Daily sitting time and long sitting bouts (bout length 20 min) both significantly decreased from baseline to the final 3-month visit (Figure 4). Average daily sitting time decreased from 645.7 (SD 72.4) min/d to 532.7 (SD 142.1; β=–112.9; *P*=.001) min/d, and average daily long sitting bouts decreased from 468.3 (SD 94.9) min/d to 366.9 (SD 150.4; β=–101.4; *P*=.002) min/d. Average daily standing time significantly increased from 219.3 (SD 63.9) min/d to 300.3 (SD 117.5; β=80.8; *P*=.005) min/d. Average daily stepping time increased from 81.3 (SD 34.3) min/d to 98.6 (SD 51.6) min/d (β=17.2, *P*=.052). Neither sit-to-stand transitions (β=–3.4; *P*=.13) nor daily MVPA (β= .11; *P*=.97) significantly changed over time.

### Physical Function Outcomes

All 3 objective physical function tests showed significant improvements (Table 2). The 4-Meter Walk Test mean time decreased from 4.23 (SD 0.95) seconds to 3.61 (SD 0.53; β=–.63; *P*=.002) seconds, the TUG mean time decreased from 10.30 (SD 3.32) seconds to 8.84 (SD 1.58; β=–1.46; *P*=.003) seconds, and the 30-Second Chair Stand mean number of sit-to-stand transitions increased from 9.75 (SD 2.81) transitions to 13.20 (SD 2.53; β=3.45; *P*<.001) transitions. Participants also self-reported improvements in physical function and fatigue. Mean score of the PROMIS physical function measure increased from 44.59 (SD 4.40) to 47.12 (SD 5.68; β=2.53; *P*=.05), indicating improved physical function. Mean score of the PROMIS fatigue decreased from 52.51 (SD 10.38) to 47.73 (SD 8.43; β=–4.78; *P*=.02), indicating reductions in fatigue. However, no significant changes were reported for anxiety (β=–2.81; *P*=.17), depression (β=–.69; *P*=.61), sleep (β=–1.16; *P*=.64), or pain (β=.52; *P*=.80).

**Table 2:** Physical function outcomes.

Physical function	Baseline, mean (SD)	Final, mean (SD)	β, LMM^a^ estimated intervention effect (SE)	*P* value
4-Meter Walk Test (s)	4.2 (0.95)	3.61 (.53)	–.63 (.17)	.002
Timed Up and Go (s)	10.3 (2.32)	8.84 (1.58)	–1.46 (.42)	.003
30-Second Chair Stand (number of stands)	9.75 (2.81)	13.20 (2.53)	3.45 (.65)	<.001

^a^LMM: linear mixed model.

### Acceptability and Feedback of the Intervention

Of the 18 (90%) out of 20 participants who completed this study’s feedback questionnaire, all participants (18/18, 100%) reported they were satisfied or very satisfied with the overall intervention. The majority of the participants (17/18, 94%) were motivated or very motivated to sit less throughout the intervention. Many enjoyed the various aspects of the intervention with 1 participant noting, “coaching was terrific, especially the 1st 3 weeks breaking the old patterns.” Participants were also asked to expand on ways that this study can better address breast cancer survivors’ needs. Most participants did not have any specific suggestions; however, 1 indicated wanting “more info on how to reduce and deal with brain fog.”

Regarding the total number of coaching sessions, of the 18 participants, 14 (78%) thought it was just the right amount while 2 (11%) reported they were a few too many sessions and 2 (11%) reported there were not enough sessions. For the first remote session, 8 were conducted via phone and 10 via Zoom. For the second remote session, 9 were conducted via phone and 9 via zoom. Of the 18 participants who responded, 11 (61%) found the number of in-person versus Zoom or phone sessions to be the right amount while 6 (33%) would have liked less in-person and more Zoom or phone sessions and 1 (6%) would have liked more in-person and less Zoom or phone sessions. When asked to expand on the improvements to the program 1 participant indicated, “I would have liked the program to last longer,” while another expressed, “Probably less in person visit and more zoom call.”

A variety of tools were offered to the participants to support behavior change. Some of the tools were used in-session with their health coach (ie, goal setting with coach, goal tracking log, personalized graph of sitting time, and the workbook) and some for use on their own outside of sessions (ie, wrist device, manual timer, and standing desk or tray). All participants (18/18, 100%) indicated that the personalized graphs were quite or extremely helpful. All participants also reported that at least 1 of the in-session or at home intervention tools listed above were helpful or extremely helpful with achieving their goal to sit less. For example, 1 participant shared that the personalized activPAL graphs of siting time were the most helpful part of the intervention: “The personalized graphs are a big stimulus to keep working on reducing sitting time. Understanding the energizing feeling by sitting less.” A few participants expressed that they would have liked to have worn the activPAL device more frequently because of the personalized graphs. Feedback on tools used outside of sessions were more mixed with the standing desk or tray being the most helpful and wrist device the least helpful. As there is no currently available wrist device that can detect sitting time, participants found the device used to be inaccurate and reported, “Sometimes it was beeping to [tell me to] move when I was moving.” However, participants still expressed interest in using a wrist device with the suggestion, “I would try to find a better wrist device!” Several participants identified joint pain as a challenge they experienced in trying to change their behavior. However, they also shared some of the benefits they felt from sitting less, including “Standing more, I discovered help me be more steady on my feet. I like that!”

Overall, the feedback from participants on the intervention was very positive. In total, 12 (67%) of the 18 participants reported they were very likely to continue to work on reducing sitting time, 5 (28%) were somewhat likely to continue reducing sitting time, and 1 (6%) indicated that they were very unlikely to continue working on reducing sitting time. For example, 1 participant stated, “I’m so glad I was asked to participate in this study and can’t wait to tell my Oncologist about it.”

## Discussion

### Principal Findings

This study highlights the feasibility of retention and adherence in a 3-month sedentary behavior intervention to reduce sitting time in breast cancer survivors. Retention and adherence were extremely high, with all 20 (100%) participants attending all 7 health coaching sessions and the final 3-month assessment. The intervention was associated with decreased sedentary behaviors, an improvement in objective and subjective measures of physical function, and decreased self-reported fatigue. Feedback from the participants indicated high acceptability with all participants who responded (18/18, 100%) indicating they were satisfied or very satisfied with Rise and found the personalized graphs helpful in changing their sitting habits.

Despite having a highly intensive in-person intervention, this study’s retention and adherence rates were very high with 100% (n=20) adherence and 0% (n=0) attrition. Although participants were very adherent, feedback highlighted participants’ desire to have fewer in-person sessions and to use more frequent remote coaching, via Zoom or phone. The high retention and adherence may have been related to our participants being highly educated and being on average more than 5 years after diagnosis [59]. However, previous research in patients with cancer has found that lower physical function contributed to higher attrition rates [59,60]. Our focus on a simple and feasible behavioral target may have supported retention in the current trial despite reported lower levels of physical functioning.

Sedentary behaviors of daily sitting time and long bouts of sitting significantly decreased by over 100 min/d on average. These significant changes are consistent, but slightly higher, than other 3-month sedentary behavior interventions which showed objective decreases in daily sedentary behaviors ranging from 36.6-72.2 min/d [26,61-63]. Participants did not significantly change sit-to-stand transitions, stepping time, nor MVPA, which is inconsistent with other studies [26,62,63]. Unlike the other studies, our intervention only focused on sitting time and did not include information on behavioral targets for any of these other behaviors. Our intervention focusing on sitting less and only impacting sitting time is consistent with previous research that has shown the distinct nature of different sedentary behaviors and the need to specifically target different behaviors, such as sit-to-stand transitions in order to change them [64]. Importantly, the lack of significant changes in stepping time and MVPA suggest that the benefits participants experienced over the 3-month intervention were not due to changes in physical activity but may have been due to reducing sitting time. This strengthens the support for focusing on sitting time to improve cancer survivorship.

Key findings of this study were that physical function and fatigue significantly improved. The improvement in physical function is consistent with sedentary behavior interventions in older adults without cancer [24,25]. As cancer survivors experience faster declines in physical function than their noncancer counterparts [6,65-67], these promising findings bolster support for targeting sedentary time in behavioral interventions for cancer survivors. The relationship between fatigue and sedentary behaviors has not been consistent across studies [7,68,69]. However, our results align with a study of objectively assessed sedentary time that found associations with improved fatigue duration at a 6-month follow-up [10]. It is important to note that the improvements in physical function and fatigue occurred without a concurrent increase in MVPA, suggesting that a sedentary behavior intervention can be effective without requiring patients to exercise. Furthermore, the use of objective measures in addition to patient-reported outcomes [70] adds to the dearth of literature surrounding sedentary behavior and breast cancer survivors.

Feedback from our multipronged intervention had overall high acceptability of the wearable devices and intervention materials. The multiple behavior components is consistent with prior studies, including our own work, suggesting that in addition to providing a device, accountability and feedback regarding the wearable tracker data are critical to the success of physical activity interventions [26,29,63,71,72]. While participants liked the thigh-worn activPAL and the accuracy of those graphs and devices, they did not like the wrist-worn tracker used for prompting standing. There were consistent recommendations for finding a more accurate wrist device. Unfortunately, existing commercial devices such as Fitbit (Fitbit LLC) and Apple Watch (Apple Inc) use the lack of steps to trigger alerts to stand, similar to the devices used for this study, and would have similar issues of incorrect alerts. With greater attention on the ill effects of sedentary behavior, we hope that future wearable devices will have better technology for identifying prolonged sitting as the likability of a wearable device has been found to increase adherence and usage in cancer survivors [73]. Finally, participants enjoyed the overall number of sessions but suggested an increased ratio of remote to in-person sessions. These reflections support changes seen across health behavior interventions as the COVID-19 pandemic has created opportunities for increased uptake and acceptability of remote care delivery among cancer survivors [74].

### Limitations

Although this was designed as a feasibility pilot study, important limitations to the findings include the small sample size, participants may not be representative of the broader breast cancer population, the lack of a control arm, and the short intervention period. As this was a pilot study, we did not control for multicomparisons in determining statistical significance. The use of multiple intervention components makes us unable to determine what aspects were most effective for behavior change. Future trials using a multiphase optimization strategy framework is important for supporting effective and cost-effective strategies to support uptake and maintenance of sitting less. Despite these limitations, this study also includes several strengths, including the use of objective measures of sedentary behaviors and physical function, being one of the first studies for breast cancer survivors to focus solely on sedentary behavior (without an exercise component), enrolled participants with low to average physical function, and had 100% (n=20) compliance and 0% (n=0) attrition over a 3-month period. The results provide important and necessary feasibility data for a future trial to assess the efficacy of the Rise intervention in an adequately powered study.

### Conclusions

Sedentary behavior interventions may support improved physical function among breast cancer survivors. In particular, the focus on solely decreasing sitting time without changes in MVPA is highly promising for the many breast cancer survivors who cannot safely or feasibly increase MVPA. These pilot results provide support for an adequately powered and longer trial. Future iterations of the intervention should include more remote and less in-person sessions, more accurate sedentary behavior wearable trackers, and assess maintenance of sedentary behavior change beyond the intervention period. Given the rapidly growing rates of breast cancer survivors in the US, the use of wearable technology and continued development of low-barrier sedentary behavior interventions is crucial in improving overall quality of life in cancer survivors.

## Abbreviations

CONSORT: Consolidated Standards of Reporting Trials
HIPAA: Health Insurance Portability and Accountability Act
LMM: linear mixed model
MVPA: moderate to vigorous physical activity
PROMIS: Patient-Reported Outcomes Measurement Information System
REDCap: Research Electronic Data Capture
TUG: Timed Up and Go

## Data Availability

The data sets generated or analyzed during this study are available from the corresponding author on reasonable request.
